# Xiaodong Zou answers questions about 15 years of research on covalent organic frameworks

**DOI:** 10.1038/s41467-020-19300-z

**Published:** 2020-11-16

## Abstract

Professor Xiaodong Zou is a full professor and chair of Inorganic and Structural Chemistry and deputy head of the Department of Materials and Environmental Chemistry at Stockholm University. After completing her PhD in structural chemistry and electron crystallography, she started at the beginning of her independent career to apply these techniques in solving structures of porous materials. She first worked on development of inorganic porous materials, such as zeolites and related open-frameworks. A joint project with Prof. Michael O’Keeffe drew her attention to metal organic frameworks (MOFs), covalent organic frameworks (COFs) and reticular synthesis. Since then, she is working on developing structure characterization techniques for porous materials.

Tell us a little bit about your research background and how you became interested in the chemistry of porous materials and COFs.

My research background is structural chemistry and crystallography. More than 90% of atomic structures were solved using X-ray diffraction. This requires growth of large crystals which is actually very difficult in many cases. Electron crystallography could be used for studying very tiny crystals. However, it was not well developed when I started my PhD in this field more than 30 years ago. We developed methods and imaging processing software for structure determination using electron crystallography. After my PhD I was looking into areas where I could apply our techniques. I became interested in research on porous materials because it was the field which really needed these techniques. There are many new and interesting materials with complicated structures developed in this field that could not be solved by X-ray diffraction. In order to understand the properties and to develop the applications further, it is important to know the structures. I first worked on zeolites and related inorganic open-frameworks, and later on MOFs and COFs. We solved the structures of many porous materials that remained unknown for years or decades. It is really rewarding to be the first person to find out the structure and how the channels and pores look like. I started to be interested in COFs when I visited Prof. Michael O’Keeffe at Arizona State University in 2005. He introduced me to reticular chemistry and I loved this design concept. O’Keeffe predicted a series of carbon-based crystalline porous materials based on reticular chemistry, and encouraged me to try to synthesize them. Unfortunately we could not get any crystalline materials. Therefore I became more interested in developing structure characterization methods to help people who are developing new porous materials. Most of my work since then has been focused on method development and structure determination.

Looking back on 15 years research on COFs, what were the highlights in COF research and which expectations remained unfulfilled?

There have been many important developments during these 15 years. One highlight is the discovery of new reactions that can link different organic building blocks to form porous crystalline COFs. From the first COFs synthesized based on condensation of boronic acids, new COFs have been developed in many different chemical systems using other condensation reactions. It shows that this is a general approach that can be widely applied. Understanding the importance of reversible bond formation has been the key for synthesizing new COFs and improving their crystallinity. Of course, with all these developments of new COFs, a challenge has been how to determine their structures. Most COF structures are deduced by combining powder XRD and model building, where many important structure details are unattainable, such as atomic positions, bond connectivity and bond nature, locations of guest species. Recently several groups succeeded in growing larger crystals of COFs by slowing down the crystallization process. This is also an important highlight. With large crystals, the missing structure details of COFs could be obtained by high resolution single crystal X-ray diffraction. This improves our understanding on how a real COF material looks like. Electron crystallography had also made a big impact on COF structural studies, single crystal structures of several COFs have been determined by 3D electron diffraction from nano- and micro-metre sized crystals.

There are many studies on applications of COFs such as gas storage and separation, catalysis, and sensing. COFs have shown large potential for applications in many areas, but much more work needs to be done in order to identify which areas will be the most promising ones for COFs.

In your opinion, which challenges need to be tackled in order to move the field further?

I think structure characterization is one of the challenges. Although there have been progresses in making large single crystals, most COF materials are still synthesized as polycrystalline powders. There is a need to develop methods which allow us to perform phase analysis and structure characterization of such powder materials with high throughputs. This would greatly help the COF community to develop the material further. We have been developing automated electron diffraction methods to study individual small particles in a powder and determine their structures. We are also developing electron diffraction methods for fast screening of the particles in a TEM and perform high throughput phase analysis of the materials. These methods can be helpful to understand crystallization processes of COFs, have a better control of the synthesis and subsequently improve the crystallinity of the materials. There are of course many other challenges in terms of synthesis and applications. We need developments in different areas, such as synthesis, characterization, modelling and application in order to push the field further.

What needs to be done in order to make COFs more widely adopted and to get a foothold in industry and industrial applications?

I think there are two things important: One is to identify key applications. Now there are many researchers exploring different applications on the academic level. If we can find key applications which are unique for COFs, they would become interesting for industry. The other is to make the COF synthesis scalable and COF materials processable. For industrial applications, cost is also an important issue. Research on COFs is still in its infancy. COFs have many unique features compared to other porous materials. It will move into industry once we find a unique application, although this may take time.

Where do you see the field going next?

The field is going towards materials engineering and applications. We need more people to explore the potential of different applications of COFs. Different applications require different materials, thus development of new materials is also important. The field also needs developments of new characterization methods. I hope one day in the future we will have tools to visualize a COF material at the atomic scale regardless of whether it is crystalline or amorphous, to see where the atoms are positioned, how the channels look like and how guest molecules interact with the framework.

*The interview was conducted by Senior Editor Dr. Johannes Kreutzer*.

